# The Effect of Coffee on Pharmacokinetic Properties of Drugs : A Review

**DOI:** 10.1155/2020/7909703

**Published:** 2020-07-24

**Authors:** Anteneh Belayneh, Fantahun Molla

**Affiliations:** ^1^Department of Pharmacy, College of Health Sciences, Debre Markos University, Ethiopia; ^2^Department of Pharmaceutics, School of Pharmacy, College of Health Sciences, Mekelle University, Ethiopia

## Abstract

**Background:**

Coffee has been the most commercialized food product and most widely consumed stimulant beverage in the world. It is a major source of caffeine which is the most bioactive component of coffee. Although both the United States Department of Agriculture and European Food Safety Authority consider daily intake of coffee which contains 400 mg of caffeine as safe for health, it causes different clinically significant pharmacokinetic interactions with many drugs. The aim of this work was to review the effect of coffee on the pharmacokinetic properties of drug**s.**

**Method:**

This review was done by investigating the *in vitro* and *in vivo* research findings, clinical case reports, and expert panels from credible sources including Scopus, PubMed, Hindawi, OVID, Google Scholar, Embase, Cochrane Library, and Web of Science.

**Result:**

Several studies and medical case reports evidently showed that concomitant consumption of coffee significantly affects the absorption, distribution, metabolism, and excretion of many drugs. These effects of coffee on the pharmacokinetics of drugs could lead to enhanced therapeutic response, therapeutic failure, or toxic reactions. *Conclusion and Recommendation*. Concomitant use of coffee should be avoided with medications which have a significant interaction with coffee. There should be an appropriate time gap between intake of drugs and coffee based on drug properties. Pharmacists and clinicians should be aware of the potential risks of drug-coffee interaction and advice patients appropriately. Further *in vitro* and *in vivo* studies should be done for frequently prescribed drugs to get a strong evidence on the pharmacokinetic interaction with coffee.

## 1. Introduction

### 1.1. Coffee

Coffee is the most commercialized food product and widely consumed beverage in the world. About 80% of the world's population consumes coffee and other coffee product each day, and this number goes up to 90% for adults in North America [1].

The worldwide coffee production reached more than 8.1 million tons per year in 2015. This accounts for more than 500 billion cups of coffee [2]. Coffee plant groups to the family Rubiaceae and genus *Coffea*. Although there are more than 80 species of coffee identified worldwide, *Coffea arabica* and *Coffea canephora* are the only two economically important species [3]. More than 70% of the global coffee market is covered by *Coffea arabica*, also known as Arabica coffee. The rest of the amount of coffee consumption is from *Coffea canephora* or Robusta coffee (commercial name of one of the main *C*. *canephora* cultivars). Coffee started its journey at least 1200 years ago in Ethiopia, then followed by Yemeni Sufi monasteries in the Middle East and northern Africa, Europe, America, and so on [4].

Coffee is composed of different chemical constituents as shown in Table 1. From all these components, caffeine, chlorogenic acids, diterpenes, and trigonelline are the most bioactive and most important contributors to the beverage flavor after roasting [2].

**Figure 1:**
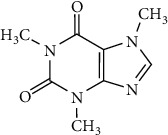
Chemical structure of caffeine [2].

Caffeine, chemically a methylxanthine (Figure 1), is a natural alkaloid and the most known bioactive component of coffee. It has a bitter taste, contributing about 10% of bitterness in the coffee beverage. As it antagonizes an adenosine receptor, caffeine stimulates the central nervous system (CNS). It was isolated from different types of coffee beans in 1820 and since then it is highly consumed in several types of food and beverages [4].

Consuming about 400 mg of caffeine (accounts for 2 to 3 cups of coffee) per day is considered safe by the US Department of Agriculture (USDA) as well as the European Food Safety Authority (EFSA) [6, 7]. Excessive caffeine intake is especially prevalent in psychiatric patients as they are frequently exposed to depression. According to Carrillo and Benitez (2000), about 22% of psychiatric inpatients were using more than 750 mg/day compared with 9% of the general population [8].

Trigonelline is also one of the chemical components of coffee which is biologically found from enzymatic methylation of nicotinic acid [7]. It is responsible for 3.5% of the bitterness of coffee. It also acts as a precursor for the formation of different groups of volatile compounds during the roasting of coffee beans. The other component responsible for the astringency, bitterness, and acidity nature of the coffee brew is chlorogenic acids [3].

## 2. Materials and Methods

### 2.1. Search Strategy

The main source for this review was electronic databases of published scientific literatures. The *in vitro* and *in vivo* research findings and clinical case reports were searched from credible sources including Scopus, PubMed, Hindawi, OVID, Google Scholar, Embase, Cochrane Library, and Web of Science. Some studies were also identified with a manual Google search. No restriction was applied to the year of publication, methodology, or study subjects. Primary search terms were “Coffee”, “Caffeine”, “Pharmacokinetic” and “Drug”.

### 2.2. Inclusion/Exclusion Criteria

Studies and medical case reports which do not contain information about the effects of coffee on the absorption, distribution, metabolism, and excretion of drugs were excluded. Articles which are published in predator journals were also excluded from this review.

## 3. Effect of Coffee on Pharmacokinetics of Drugs

The pharmacokinetics of drugs is affected by different factors via different mechanisms such as altering the absorption, distribution, excretion, and induction or inhibition of metabolizing enzymes [9]. One of the major factors which affect the pharmacokinetic property of drugs is beverage. Coffee is one of the most known beverages which have a pharmacokinetic interaction with many drugs [10, 11].

Patients as well as healthy people throughout the world are starting and finishing their days with cups of coffee [12]. Even though coffee has numerous benefits to our body, there is also a clinically significant pharmacokinetic interaction between coffee and many important over-the-counter as well as prescription medications due to its major constituents, mainly caffeine and chlorogenic acid [8]. The aim of this work was to review the effect of coffee on the absorption, distribution, and elimination of some drugs.

### 3.1. The Effect of Coffee on Absorption of Drugs

Although the oral route has several advantages, the pharmacokinetic properties of drugs administered in this route are affected by the presence of food products such as coffee and other caffeinated products [9].

Coffee can affect the absorption process of drugs by changing the dissolution profile, changing the gastrointestinal (GI) pH, affecting the sink condition of the GI membrane and blood, affecting the GI emptying time, formation of complex, and inhibiting glucose-6-phosphatase.

#### 3.1.1. Effect on Absorption by Complex Formation

**Figure 2:**
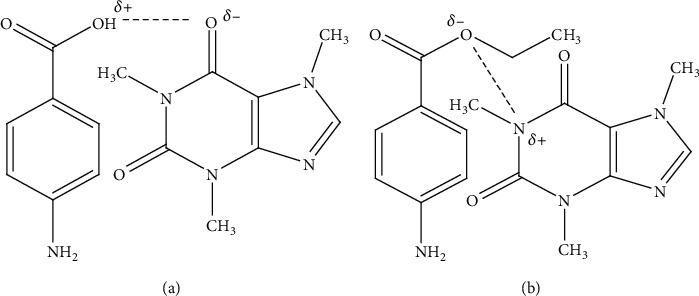
Complexation mechanism of caffeine with drugs by hydrogen bond (a) and dipole–dipole force (b) [15].

Caffeine, the main chemical component of coffee, is responsible for the complex formation with different chemicals. Caffeine interacts with different acidic drugs. This interaction occurs by a dipole–dipole force or hydrogen bonding between the polarized carbonyl groups of caffeine and the hydrogen atom of these acidic drugs as shown in Figure 2 [13]. It forms more soluble complexes with several drugs which have organic acid anions. But, sometimes less soluble complexes of drugs with some other organic acids such as gentisic acid are formed. Caffeine forms hydrogen-bonded complexes with various drugs which have a proton donor functional group: phenol, phenol derivatives, and aliphatic alcohols. For instance, with the addition of caffeine, the solubility of p-aminobenzoic acid (PABA) rises linearly due to complexation [14].

Coffee and other caffeine-containing products are found to interfere with the pharmacokinetic profile of escitalopram oxalate (selective serotonin reuptake inhibitor). The *in vitro* study on the release showed that the absorption of escitalopram oxalate decreased by the formation of complexes between the drug and caffeine. This complex formation reduced the bioavailability of escitalopram oxalate tablets by 33.15% (*in vitro*) and 28% (*in vivo*) by decreasing the dissolution of the escitalopram tablet [11].

The intestinal absorption of both inorganic and organic compounds could be decreased by coffee. For instance, about 39 to 90% of iron absorption is reduced when a cup of coffee as well as other caffeinated beverages is taken with an iron rich meal. Conversely, there was no decrease in iron absorption when coffee was consumed one or two hours before a meal [16]. Due to this, coffee and other beverages containing caffeine should be separated from iron-containing foods or supplements by at least one hour [14].

**Figure 3:**
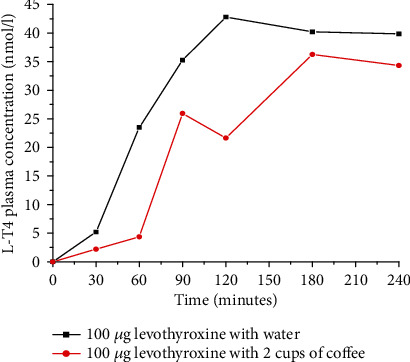
Comparing serum thyroxine (T4) level after ingestion of 100 *μ*g levothyroxine (L-T4) with water and with 2 cups of coffee (espresso) in healthy volunteers (redrawn from published paper [6]).

Patient case reports in Messina, Italy, showed that coffee also reduces the intestinal absorption of thyroxine 4 (T4) by 55%. This is due to coffee sequestering T4 and rendering fewer hormones available for the uptake by the intestinal epithelium as shown in Figure 3. As a result, coffee should be added in the list of interferers of T4 intestinal absorption by drug regulatory bodies. By the same token, T4 should be added to the list of compounds whose absorption is affected by coffee. The serum concentration of T4 was measured with the chemiluminescent assay by Roche Diagnostics (Italy) [6].

A case report of a 52-year-old white woman in Albany, New York, also showed that the absorption of L-T4 was significantly decreased due to routine ingestion of L-T4 with a daily cup of coffee. The authors observed that increased thyroid-stimulating hormone (TSH) secretion was a feedback of lowering L-T4 concentration in the blood due to routine coffee ingestion. Coffee should be uniformly added to the list of compounds that may decrease L-T4 absorption across drug compendia [17].

#### 3.1.2. Effect on Absorption by Changing the Gastrointestinal pH

Oral and gastric bitter taste receptors are involved in the regulation of gastric acid secretion in humans. Bitter tastants elicit bitterness through a family of oral taste type 2 bitter receptors (TAS2Rs). Caffeine is a known stimulant of gastric acid secretion (GAS) by activating several taste type 2 bitter receptors (TAS2Rs) due to its bitter taste. This leads to a decrease in the gastric pH by increasing the hydrochloric acid secretion. The production of excess hydrochloric acid in the stomach can affect the extent of drug absorption through acid degradation of the drug, conversion of the drug substance into an insoluble form, and by altering the drug's rate of dissolution [13]. The rate of drug degradation by the acid catalyzed process is pH dependent; thus, a little change in pH can have a great impact on the amount of drug that survives gastric transit. In addition to its acidic nature, coffee also stimulates the hypersecretion of hydrochloric acid in the stomach. As a result, this highly acidic stomach contents are easily passed into the small intestine more rapidly than normal. This may lead to a decrease in the pH of the intestinal environment which affects the absorption of several basic drugs [18]. For example, a drop of one pH unit reduces the amount of active midazolam by 75% after drinking two to three cups of coffee. Generally, increased gastric acid secretions in the stomach results to a faster dissolution of basic drugs which makes the absorption rate of such drugs by twenty times faster [19].

*In vitro* study showed that the addition of coffee to neuroleptic drugs such as phenothiazine (including fluphenazine, chlorpromazine, thioridazine, prochlorperazine, and trifluoperazine) and butyrphenone (antipsychotic drug) forms an insoluble precipitant. This coffee–neuroleptic drug precipitation reaction has a great clinical significance by decreasing the absorption of those neuroleptic drugs [20].

The proportion of chlorpromazine (CPZ) precipitated by a cup of coffee was 80% at low doses of the drug (10-40 mg). In case of high doses (800 mg) of CPZ, the proportion of the drug precipitated was about 20% [20]. *In vivo* studies by the oral administration of coffee and CPZ showed that the cataleptic effect of CPZ is significantly reduced due to the caffeine present in coffee. This interaction might also affect the absorption of orally administered phenothiazines [21].

**Figure 4:**
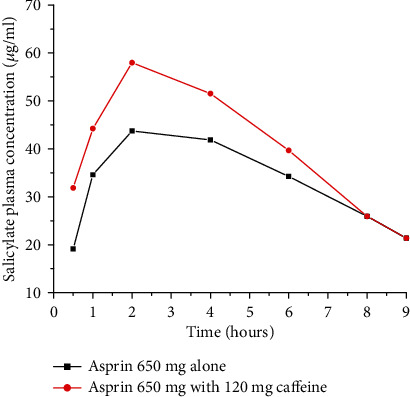
Plasma salicylate concentration-time curve after oral administration of 650 mg aspirin alone or in combination with coffee (120 mg caffeine) in a human volunteer study (redrawn from published paper [24]).

The ingestion of two cups of coffee (equivalent to 120 mg caffeine) along with 650 mg of aspirin (NSAID) increased the rate of aspirin absorption significantly [22, 23]. The peak plasma level and the bioavailability of aspirin increased without affecting drug elimination as shown in Figure 4. The mechanism by which caffeine affects the rate of aspirin absorption is by enhancing the gastric acid secretion. The resulting reduction in gastric pH may have increased the unionized form of the drug and facilitates its absorption. Moreover, caffeine is known to increase the microcirculation in the gastric mucosa probably by increasing the level of cAMP; this might also contribute to the higher absorption [24].

The solubility of halofantrine (important basic drug in malaria treatment) is enhanced by coffee and other drinks that contain different amounts of caffeine. It is evident that coffee increases the *in vitro* solubility of basic drugs such as halofantrine markedly by decreasing the gastrointestinal pH. Weakly basic drugs dissolve better in the presence of an acidic pH as it forms a soluble salt [12].

**Figure 5:**
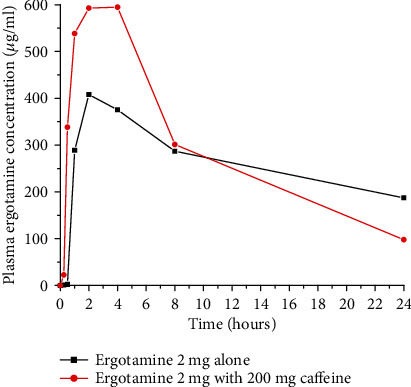
Plasma concentration-time curve after administration of 2 mg ergotamine tartrate alone or in combination with 200 mg caffeine in rats (redrawn from published paper [23]).

Caffeine enhances the bioavailability of ergotamine, which is an important ergot alkaloid derivative drug for migraine treatment [25]. A possible explanation for the quicker onset of action and increase extent would be due to faster and more complete gastrointestinal absorption of ergotamine in the presence of caffeine as shown in Figure 5 [23]. Movement of the drug molecules from a lipid phase (gastrointestinal membrane) into an aqueous phase (blood) is the rate-determining step of ergotamine absorption. Caffeine can accelerate the absorption rate of ergotamine by enhancing its aqueous solubility through reducing gastric pH [26].

**Figure 6:**
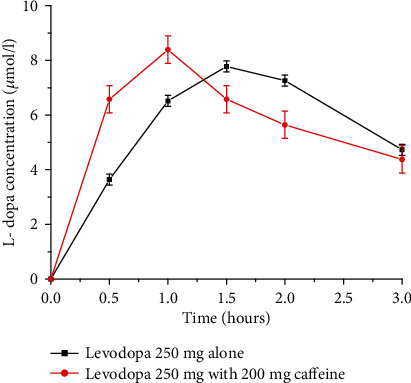
Levodopa plasma concentration profile after administration of a single oral dose of levodopa 250 mg alone and with caffeine 200 mg in human volunteers (Redrawn from published paper [25]).

Caffeine accelerates the levodopa (anti-Parkinson drug) uptake which significantly contributes in shortening of the latency to motor response. A possible mechanism for decreasing *T*_max_ could be due to reduced gastric pH which in turn may enhance gastric emptying and consequently leads to faster levodopa absorption. The pharmacokinetics of levodopa after caffeine and placebo intake is shown in Figure 6. The *T*_max_ for levodopa is shorter with caffeine than with placebo (1 hour versus 1.5 hours). However, levodopa's *C*_max_ and AUC values are comparable for both cases [25].

**Figure 7:**
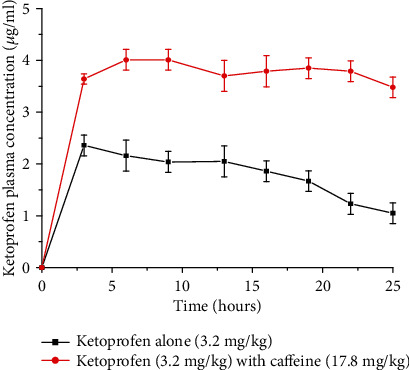
Variation of the pharmacokinetic profile when orally administered with ketoprofen alone (3.2 mg/kg) and ketoprofen administered with caffeine (17.8 mg/kg) in rats (redrawn from published paper [27]).

The pharmacokinetics of ketoprofen (NSAID) alone and in combination with caffeine has been determined in one recent study. Accordingly, after administration of a single dose of ketoprofen alone or ketoprofen plus caffeine to rats, there were significant differences in *C*_max_ and AUC of ketoprofen as shown in Figure 7. When ketoprofen is administered with caffeine, the values of *C*_max_ and AUC were increased by 90.1% and 82.7%, respectively. The ketoprofen plasma concentrations were determined by HPLC analysis with ultraviolet (UV) detection [27].

A preclinical study by Raul et al. (2018) also showed significant differences in *C*_max_, AUC_0-24_, and AUC_0-∞_ values of ketoprofen with caffeine administration. However, the authors concluded that the exact pharmacokinetic mechanism of how caffeine increases the plasma level of ketoprofen is still not clear. The most probable reason, according to the authors, is due to decreasing gastric pH, reducing gastric emptying, and increasing blood flow of the gastric mucosa effects of caffeine. In addition, coffee decreases the metabolic clearance of ketoprofen by decreasing the liver blood flow [27].

A study done on voluntary humans in Malaysia showed that there was an increase in the paracetamol rate and extent of absorption with coffee containing 65 and 195 mg of caffeine. The *T*_max_ of paracetamol was decreased by about 48% on concomitant administration of caffeine, and the *C*_max_ of paracetamol was increased by 7.6% and 10.6% with 65 mg and 195 mg caffeine doses, respectively. Moreover, the AUC of paracetamol was increased by 6.92% and 43.33% with 65 mg and 195 mg caffeine, respectively. The observed mean peak paracetamol serum concentration increase, however, was within the therapeutic range of paracetamol (10-20 *μ*g/ml). Therefore, it suggests that paracetamol toxicity will not occur even when it was coadministered with coffee [28].

Another study done in Germany on 24 health volunteers also showed that caffeine accelerated paracetamol absorption, indicated by enhanced early AUCs. Th*e in vitro* dissolution tests revealed that this accelerated absorption was due to a faster dissolution rate of paracetamol occurring during the coadministration of caffeine compared to the reference paracetamol standard formulation [29].

#### 3.1.3. Effect on Absorption by Changing Gastric Emptying Time

Coffee can also affect the absorption of some drugs by affecting the gastric emptying time. It increases the absorption rate of drugs by fastening the gastric emptying rate and makes the drug available for absorption faster [8, 30]. Nevertheless, coffee does not have any significant effect on the gastric emptying of liquid medications [31].

Aspirin deliver its analgesic effect faster and more effectively when administered with coffee or coformulated with caffeine. This might be due to caffeine's tendency to increase the gastric emptying rate. Faster gastric emptying rate makes the drug available for absorption sooner. In addition, aspirin undergoes a rapid hydrolysis to salicylic and acetic acid in the stomach and thus shorter gastric residence time reduces the amount of hydrolysis that occurs prior to absorption [11]. Likewise, drugs that have been administered as salt form generally convert rapidly to the less soluble parent form in the stomach. For these drugs, coadministration with coffee may limit the time available for conversion which results in higher systemic exposure than if the drugs were administered alone [19].

A human study done in Canada showed that the plasma concentration of antihypertensive drug felodipine was significantly increased when taken with caffeine. Both the AUC and *C*_max_ of felodipine were increased from 8.66 to 10.57(ng h/ml) and 1.65 to 2.10 ng/ml due to caffeine, respectively. This enhanced absorption of felodipine might be due to fastening of the gastric emptying rate by caffeine which makes the drug available for absorption faster [32].

#### 3.1.4. Effect on Absorption by Affecting Sink Condition

Coffee inhibits vitamin D receptors on osteoblasts, the cells responsible for producing the bone, which limit the amount that will be absorbed. The cross-sectional study done on randomly selected 330 Saudi boys and girls aged 11–14 years showed that vitamin D levels of the blood were significantly elevated among those consuming 9–12 cups of coffee/week [33]. When vitamin D receptors are inhibited, the vitamin D concentration in the blood is high. If the concentration of vitamin D in the blood is high, then the absorption into osteoblasts will be reduced highly because of offsetting sink condition, i.e., the driving force, concentration gradient, is nullified [23]. As vitamin D is important in the absorption and use of calcium in building the bone; this might cause a decrease in the bone mineral density which results in an increased risk for osteoporosis [14]. This case is more pronounced in elder populations since they have a reduced capacity to synthesize vitamin D receptors [34].

Coffee also decreases the efficiency of certain drugs whose absorption is highly affected by vitamin D such as calcium and zinc. Because calcium absorption is directly related to vitamin D absorption, coffee also decreases the efficiency of calcium absorption [18, 34]. One study shows that women who consumed more than two cups of coffee per day had a risk of fracture over the next 12 years, which was 69% higher than women who did not consume coffee and caffeinated beverages. Coffee also decreases the absorption of the antiosteoporosis drug alendronate. Taking alendronate with coffee reduces its absorption by about 60%. The drug needs to be taken with water on an empty stomach at least 30 minutes before coffee to get an optimum pharmacological effect [14].

#### 3.1.5. Effect on Absorption by Inhibiting Glucose-6-Phosphatase

Coffee may also decrease the intestinal absorption of glucose. The responsible constituent of coffee for this action is chlorogenic acid (CGA). CGA, one of the most abundant polyphenol compounds in coffee, might decrease the absorption of glucose by inhibiting the activity of glucose-6-phosphatase which is responsible for the release of glucose into the general circulation. A study which was conducted on 12 healthy volunteers over a 120 min study period revealed that the AUC of glucose was significantly decreased by simultaneous ingestion of coffee [35]. Another study done in Japan on volunteer men also showed that the concomitant consumption of coffee showed statistically significant decreases in the 2-hour concentrations and the area under the curve of glucose at 16 weeks. Coffee decreases the mean percent concentration of glucose in 2 hours and the AUC of glucose in 16 weeks by 13.1% and 7.5%, respectively. This is may be due to a competitive inhibition of glucose metabolizing enzymes by coffee [36].

Coffee also reduces the intestinal absorption of glucose by encouraging the dispersal of the Na+ electrochemical gradient which draws glucose into the enterocytes [37].

### 3.2. The Effect of Coffee on Distribution of Drugs

After absorption from different sites of administration or systemic administration directly into the bloodstream, a drug distributes into the interstitial and intracellular fluids of the whole-body part. This process involves passing of a number of physiological barriers which partly depends on physicochemical properties of the individual drug [23]. The most important physiological factors which affect drug distribution are blood-tissue partitioning and availability of different barriers which prevent the distribution of drugs. Blood-tissue partitioning refers to the relative binding ability of a drug to plasma proteins and tissue macromolecules that limits the concentration of free drug. Blood-brain barrier is the most common barrier which affects drug distribution to the central nervous system [9].

#### 3.2.1. Effect of Coffee on Distribution by Enhancing the Tightness of Blood-Brain Barrier

A clinical study by John (2015) showed that coffee protects the blood-brain barrier (BBB) breakdown by maintaining the expression levels of tight junction proteins. These proteins bind the cells of the BBB tightly to each other to prevent unwanted molecules, even highly lipophilic drugs, crossing into the central nervous system [19]. The stabilization of BBB by coffee has great implications during therapeutic interventions of neurological disorders in two ways. First, caffeine can prevent the opening of the BBB which might hinder the passage of important drugs into CNS including drugs with high lipophilic nature. Because of this, it is important to limit ingestion of coffee by patients taking drugs acting in the CNS; for instance, drugs used for Alzheimer treatment such as memantine and donepezil. On the other hand, coffee can prevent the passage of different toxic substances to CNS by strengthening the junction of the BBB [38].

#### 3.2.2. Effect of Coffee on Distribution by Inhibiting Metabolizing Enzyme at Peripheral NS and CNS

**Figure 8:**
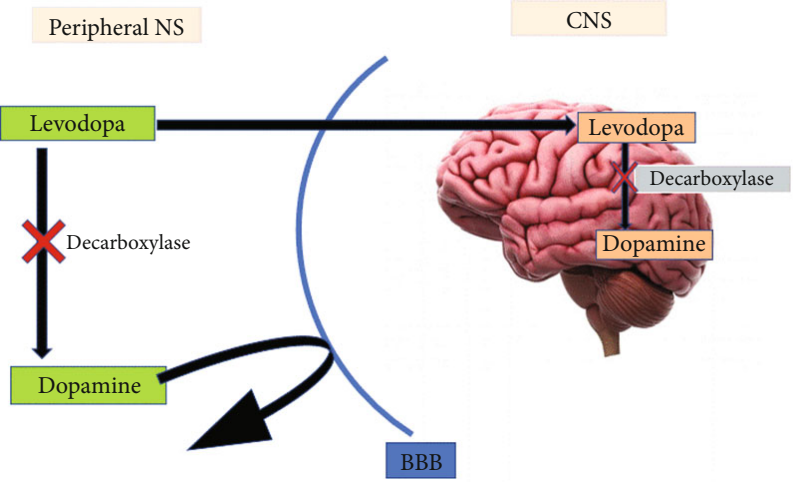
Effect of coffee on the levodopa pathway into CNS [9].

Levodopa enters the brain across the BBB by an active transport system. Unlike dopamine (DA), L-DOPA is able to cross the blood-brain barrier easily and enters the central nervous system (CNS). Peripherally and after entering the CNS, L-DOPA is converted into DA by the aromatic enzyme called L-amino acid decarboxylase [11]. The ingestion of coffee slows down the conversion of L-DOPA into DA by inhibiting the decarboxylase enzyme as shown in Figure 8. This inhibition process of coffee has two important applications: (1) it facilitates the distribution of levodopa to brain tissues by active transport and (2) it helps to extend the half-life of L-DOPA in the brain. L-DOPA has a *t*_1/2_ of 60–90 minutes, but the half-life will be more than double when it is administered after ingestion of more than 2 cups of coffee [39].

A study done in Florida, USA, also showed that coffee consumption enhances the effects of L-DOPA and has a preventive role in both the onset and progression of PD. Caffeine may behave as an allosteric or competitive inhibitor of an enzyme that participates in converting L-DOPA to DA. Coffee also has been reported to increase the plasma levels of L-DOPA. The study noted that further studies are needed to determine the long-term effects of the combination treatment and what concentrations of coffee are optimal in treating PD [40].

### 3.3. The Effect of Coffee on Metabolism of Drugs

Enzymes (mostly liver enzymes) that are secreted over time to protect the body from exogenous chemicals are responsible for most of drug metabolism reactions [23]. The metabolites are mostly less pharmacologically active or generally inert. Metabolism also usually makes the drugs more water soluble so that they can be easily excreted [9]. Coffee is one of the factors which affect drug metabolism by affecting metabolizing enzymes in different ways.

#### 3.3.1. Effect of Coffee on Metabolism by Saturating Enzymes

The dominant and most abundant enzyme that is responsible of metabolizing caffeine is a CYP450 liver enzyme also called CYP1A2. This enzyme also has a key role for the metabolism of several clinically important medications. When caffeine and drugs (that are metabolized by CYP1A2) are administered together, computing for the same enzyme is common. Consequently, the availability of enzymes to metabolize drugs is decreased as it is saturated by caffeine. In other word, caffeine and drugs act as a metabolic inhibitor to each other which lead to the decreased rate of elimination of drugs [41].

A study by John et al. (2013) on healthy volunteers showed that the plasma concentration of clozapine (antischizophrenic medication) was increased by 97% after concomitant administration of 2-3 cup of coffee (caffeine 400 mg/day). This study also showed that heavy coffee drinker women who were on clozapine medications were found to have about two and half times higher blood levels of the drug in comparison to noncoffee drinkers [19]. In addition to clozapine, the blood concentration of lithium, theophylline, warfarin, and several antidepressants and antipsychotics drugs also increased after concomitant ingestion of coffee as they are metabolized by a similar enzyme with caffeine. The consequences of these pharmacokinetic drug interactions are highly variable [20].

A study done on healthy adult male albino rabbits to assess the effect of caffeine on the anticoagulation activity of warfarin revealed that coffee significantly inhibits the metabolism of warfarin by saturating enzymes which is responsible of metabolizing it. Because of this, ingestion of caffeine increased the plasma concentration of warfarin which leads to enhanced anticoagulant effects. This result suggests that health professionals should advise patients to stop the frequent use of coffee and other caffeine-rich products during warfarin therapy [41].

There are many drugs whose rate of metabolism is inhibited by the presence of caffeine as shown in Table 2. These drugs will be metabolized more slowly and remain active in the body longer when the patient consumes coffee during the treatment period [42].

A study done in Finland on voluntary humans showed that when caffeine was administered with melatonin (drug used for sleep disorder), the *C*_max_ and AUC of melatonin were increased on average by 142% and 120%, respectively. This pronounced effect of caffeine on the bioavailability of orally given melatonin, according to the study, was most probably due to a competitive inhibition of the CYP1A2 catalyzed first-pass metabolism of melatonin [43]. Another study done in Spain also showed that the blood concentration of several drugs, including certain selective serotonin reuptake inhibitors (fluvoxamine), antiarrhythmics (mexiletine), antipsychotics (clozapine, psoralens, idrocilamide, and phenylpropanolamine), bronchodilators (furafylline and theophylline), and quinolones (enoxacin), were higher when taken with caffeine concomitantly. This might be due to the competitive inhibition of isoenzymes responsible of metabolizing those drugs by caffeine [8].

Another study done by combining the administration of 7.5 mg of zolpidem (common sleeping pill) and 250 mg caffeine to twelve healthy male and female volunteers in USA showed that caffeine coadministration modestly increased the *C*_max_ and the AUC of zolpidem by 30–40%. This increased effect of caffeine might be attributable to the competitive inhibition of CYP1A2 by caffeine which is responsible of metabolizing zolpidem [44].

An *in vitro* study in Thailand showed the metabolism of dextromethorphan (cough syrup) was significantly inhibited by caffeine (10-100 *μ*g/ml). This is due to a strong inhibition of cytochrome p450 CYP2D6 (enzyme responsible to metabolism of dextromethorphan) by caffeine. This finding suggests that when dextromethorphan and a high concentration of caffeine coexist, it produces longer and stronger effects in the human body because the unchanged form of dextromethorphan remains in the body for a longer term [45].

#### 3.3.2. Effect Coffee on Metabolism by Inducing Enzyme

**Figure 9:**
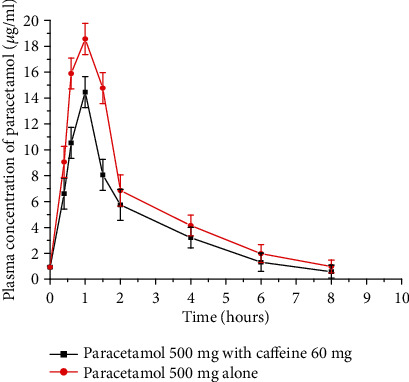
Mean plasma concentration vs. time profiles of paracetamol following the administration of 500 mg paracetamol alone and with 60 mg caffeine on healthy male volunteers (redrawn from published paper [46]).

A study done on ten healthy male human volunteers showed that paracetamol was largely cleared from the blood through biotransformation by hepatic cytochrome P-450 and P-448 mixed function oxidases. Caffeine induces the activity of cytochrome P-448 that metabolizes paracetamol. Due to its enzyme-inducing ability, caffeine can lower the concentration of the drug in the plasma, decrease the area under curve, and increase the total body clearance. The participating subjects were given 500 mg paracetamol tablet or a combination of 500 mg paracetamol and 60 mg caffeine. A reverse-phase HPLC method was used for the separation, identification, and quantification of paracetamol. As depicted in Figure 9, coffee decreased the *C*_max_ and AUC of paracetamol [46].

### 3.4. The Effect of Coffee on the Excretion of Drugs

Immediately after being absorbed into the systemic circulation, drugs are removed from the body either unchanged by the process of excretion or converted to metabolites. Most drugs and drugs metabolites are removed by the kidney as it is the most important organ for excretion [23]. Some drugs and metabolites are excreted either in the bile or secreted directly into the intestinal tract and not reabsorbed [9]. There are different mechanisms in which coffee affects the excretion of some drugs.

#### 3.4.1. Effect of Coffee on Excretion by Increasing Volume of Urine

One of the main side effects of consuming too much coffee is frequent urination. The mechanism of caffeine to increase urination is by increasing the glomerular blood pressure within capillaries in the kidney. Due to this mechanism, coffee increases the blood filtration which leads to an increase in urine formation [19]. This can cause the body to become quite easily and quickly depleted of some of the nutrient essential for maintaining good health and some medications. Minerals that can become depleted very easily include calcium, magnesium, sodium, phosphate, and potassium. A hormone called antidiuretic hormone (ADH) is responsible to regulate urine production in the body. Based on the condition of body dehydration and the need to retain water, the body releases this hormone which concentrates the urine to save body fluids. Coffee causes much production of dilute urine by inhibiting the ADH [47]. The study done in Czech Republic also showed that even moderate daily intake of coffee at the dose up to 400 mg daily may be associated with adverse outcomes, like bone status and calcium balance [48].

As one observational study on women showed that for every 150 mg of caffeine ingested (equivalent to one to two cup of coffee), there was a loss of 15 mg of calcium even hours after the consumption of caffeine. This study also showed that women who consumed more than 300 mg of caffeine had lost more bone in the spine than women who consumed less amount of caffeine [19]. In addition, more hip fractures occurred in these women than those who avoid caffeine or drink in moderation. Water-soluble vitamins such as the B vitamins (B1 and B12), magnesium, sodium, chloride, and water can be also depleted as a result of the fluid loss due to the diuretic effect of caffeine [49].

The excretion profile of oxandrolone and epioxandrolone (anabolic steroids) are also altered by coffee. A medical case report showed that the ingestion of three cups of coffee (equivalent to 300 mg of caffeine) highly increased (about 20 times) the rate and excretion of both oxandrolone and epioxandrolone. The maximum excretion rate of oxandrolone is 10 ng/min; nevertheless, it increases to 150 ng/min after concomitant ingestion of coffee [47].

There is evidence that caffeine actions are mainly mediated by antagonism of adenosine receptors. Caffeine is structurally similar to adenosine, an extracellular messenger that acts at specific cell surface receptors. Adenosine has an important role in the kidney by virtue of its mediator action in the tubule-glomerular feedback response [50]. Caffeine has been associated with increased urinary volume and frequency, causing the body to lose water and electrolytes such as potassium and sodium [15]. As caffeine oppose the vasoconstriction of renal afferent arteriole which is mediated by adenosine, the glomerular filtration rate of kidney is increased. It also inhibits the sodium reabsorption rate at the level of renal proximal tubules [51].

Generally, several *in vitro* and *in vivo* studies and clinical case reports showed that coffee and other caffeinated products affect the different pharmacokinetic properties of several drugs as summarized in Table 3.

## 4. Conclusion and Recommendation

Coffee consumption at varying levels has a significant influence on the absorption, distribution, and elimination of some drugs. These effects of coffee on the pharmacokinetics of drugs could cause enhancing therapeutic response, therapeutic failure, and/or may cause toxic reactions in patients receiving those drugs. Especially, it may cause dangerous problems when ingested with narrow therapeutic index drugs which have a pharmacokinetic interaction with caffeine. Due to this, clinicians should be aware of the potential risks of pharmacokinetic interaction between dietary coffee intakes with medications. Unless a lack of interaction has already been demonstrated for a particular drug, consumption of coffee and other caffeine-containing food and beverages should be restricted as appropriate. It is recommended that drugs interacting with coffee should be appropriately labeled. There should be also an appropriate time gap between intake of drugs and coffee based on drug properties. Drug regulatory bodies and researchers should facilitate further *in vitro* and *in vivo* studies for commonly prescribed drugs to assess pharmacokinetic interaction with coffee.

## Figures and Tables

**Table 1:** Chemical composition of a cup coffee (150 ml) made from 7.5 g of coffee powder [5].

Constitute of coffee	Amount (mg) in 1 cup of coffee
Caffeine	80-170
Protein	100
Water soluble polysaccharides	410-432
Saccharose	13.5
Monosaccharides (glucose and fructose)	7
Volatile acids (formic acid and acetic acid)	24
Nonvolatile acids (lactate, pyruvate, tartrate, and citrate)	27
Chlorogenic acids	250-292
Lipids	13.5
Trigonelline	27
Nicotinic acid	1.4
Volatile aromatic compounds	7
Minerals	240
Others	460

**Table 2:** List of drugs whose rate of metabolism is slowed by caffeine [8].

Class of drug	Generic name
Antidepressants	Amitriptyline, clomipramine, fluvoxamine, mianserin, and imipramine
Antipsychotics	Clozapine, haloperidol, and olanzapine
Cardiovascular drugs and anticoagulants	Lidocaine, mexiletine, propafenone, propranolol, triamterene, verapamil, and warfarin
Cholinesterase inhibitors	Tacrine
Local anesthetic	Ropivacaine
Nonsteroidal anti-inflammatory drugs	Methotrexate
Quinolones	Pefloxacin

**Table 3:** Summary of the effect of coffee on the pharmacokinetics of some drugs.

Drugs	The effect of coffee on the PK of drugs	Mechanism	References
Escitalopram oxalate	Decrease absorption	Insoluble complex formation	[11]
Iron and iron-containing drugs	Decrease absorption	Insoluble complex formation	[14]
Midazolam	Decrease absorption	Changing pH	[19]
Phenothiazines and butyrophenone	Decrease absorption	Changing pH	[20]
Aspirin and felodipine	Increase absorption	Changing pH, increase gastric emptying rate	[24, 32]
Halofantrine	Increase absorption	Changing pH	[12]
Ergotamine and levodopa	Increase absorption	Changing pH	[25]
Thyroxine (T4)	Decrease absorption	Sequestering	[6]
Vitamin D and calcium	Decrease absorption	Affecting sink condition	[14]
Glucose	Decrease absorption	Inhibition of the activity of glucose-6-phosphatase	[35, 36]
Memantine and donepezil	Decrease distribution	By enhancing the tightness of the blood-brain barrier	[52]
Levodopa	Increase distribution	By inhibiting a metabolizing enzyme at peripheral NS and CNS	[39]
Clozapine, amitriptyline, lithium, theophylline, warfarin, clomipramine, fluvoxamine, imipramine, haloperidol, olanzapine, lidocaine, mexiletine, propranolol, triamterene, verapamil, ropivacaine, melatonin, dextromethorphan, methotrexate, and pefloxacin	Inhibit metabolism	By saturating a metabolizing enzyme	[8, 19, 41–43, 45, 53, 54]
Paracetamol	Enhancing metabolism	Induce the activity of cytochrome P-448	[46]
Minerals (calcium, magnesium, sodium, and chloride), water-soluble vitamins (vit B), oxandrolone, and epioxandrolone	Increase excretion	Increasing volume of urine	[49, 51]

## References

[1] Dunwiddie T. V., Masino S. A. (2001). The role and regulation of adenosine in the central nervous system. *Annual Review of Neuroscience*.

[2] Chu Y. F. (2016). *Coffee: Emerging Health Effects and Disease Prevention*.

[3] Maurin O., Davis A. P., Chester M., Mvungi E. F., Jaufeerally-Fakim Y., Fay M. F. (2007). Towards a phylogeny for Coffea (Rubiaceae): identifying well-supported lineages based on nuclear and plastid DNA sequences. *Annals of Botany*.

[4] Wolf A., Bray G. A., Popkin B. M. (2008). A short history of beverages and how our body treats them. *Obesity Reviews*.

[5] Boekema P. J., Samsom M., van Berge Henegouwen G. P., Smout A. J. P. M. (2009). Coffee and gastrointestinal function: facts and fiction: a review. *Scandinavian Journal of Gastroenterology*.

[6] Benvenga S., Bartolone L., Pappalardo M. A. (2008). Altered intestinal absorption of L-thyroxine caused by coffee. *Thyroid*.

[7] Nutrition facts 2012. *Caffeine – is there a safe amount?*.

[8] Carrillo J. A., Benitez J. (2000). Clinically significant pharmacokinetic interactions between dietary caffeine and medications. *Clinical Pharmacokinetics*.

[9] Goodman G. (2012). *Goodman and Gilman's the Pharmacological Basis of Therapeutics*.

[10] Adam M. P. (2013). Foundations in Pharmacokinetics. https://uncpress.org/book/9781469636009/foundations-in-pharmacokinetics/.

[11] Krishna V., Gouthami B., Meyyanathan S., Gowramma B., Elango K., Suresh B. (2013). *In vitro In vivo* pharmacokinetic interaction study of escitalopram oxalate when co administered with caffeine/caffeinated beverages. *The Open Conference Proceedings Journal*.

[12] Babalola C. P. (2014). Effect of caffeine-containing beverages on physicochemical and release properties of halofantrine. *Global Journal of Medical Research*.

[13] Jain G., Khar R. K., Ahmad F. J. (2013). *Theory and practice of physical pharmacy-e-book*.

[14] Lippincott W., Wilkins M. (2008). Nutrition and Diagnosis-Related Care. https://books.google.com.

[15] Chen X., Ghribi O., Geiger J. D. (2010). Caffeine protects against disruptions of the blood-brain barrier in animal models of Alzheimer's and Parkinson's diseases. *Journal of Alzheimer's Disease*.

[16] Layrisse M., García-Casal M. N., Solano L. (2000). New property of vitamin A and *β*-carotene on human iron absorption: effect on phytate and polyphenols as inhibitors of iron absorption. *Archivos Latinoamericanos de Nutrición*.

[17] Wegrzyn N. M. (2016). Malabsorption of L-T4 due to drip coffee: a case report using predictors of causation. *Journal of the Academy of Nutrition and Dietetics*.

[18] Meri R., Theresa G., Gerri F. (2004). Effects of caffeine and coffee on heartburn, acid reflux, ulcers & GERD. https://healthy.net/2019/01/06/effects-of-caffeine-and-coffee-on-heartburn-acid-reflux-ulcers-and-gerdreviewed-by-meri-rafetto-rd-theresa-grumet-rd-and-gerri-french-rd-ms-cde/.

[19] Roush J. (2015). *How caffeine affects drug absorption: Vista Biopharmaceutics*.

[20] World of Caffeine (2015). The Science and Culture of the World's Most Popular Drug. http://worldofcaffeine.com/drug-and-food-interactions-with-caffeine/.

[21] Cheeseman H. J., Neal M. J. (1981). Interaction of chlorpromazine with tea and coffee. *British Journal of Clinical Pharmacology*.

[22] Yoovathaworn K. C., Sriwatanakul K., Thithapandha A. (1986). Influence of caffeine on aspirin pharmacokinetics. *European Journal of Drug Metabolism and Pharmacokinetics*.

[23] Schmidt R., Fanchamps A. (1974). Effect of caffeine on intestinal absorption of ergotamine in man. *European Journal of Clinical Pharmacology*.

[24] Craig C. R., Stitzel R. E. (2004). *Modern Pharmacology with Clinical Applications*.

[25] Deleu D., Jacob P., Chand P., Sarre S., Colwell A. (2006). Effects of caffeine on levodopa pharmacokinetics and pharmacodynamics in Parkinson disease. *Neurology*.

[26] Anderson J. R., Drehsen G., Pitman I. H. (1981). Effect of caffeine on ergotamine absorption from rat small intestine. *Journal of Pharmaceutical Sciences*.

[27] Medina-López R., Vara-Gama N., Soria-Arteche O., Moreno-Rocha L., López-Muñoz F. (2018). Pharmacokinetics and pharmacodynamics of (S)-Ketoprofen co-administered with caffeine: a preclinical study in arthritic rats. *Pharmaceutics*.

[28] Myat T., Thu W. (2019). Coffee modify pharmacokinetics of acetaminophen. *EC Pharmacology and Toxicology*.

[29] Renner B., Clarke G., Grattan T. (2007). Caffeine accelerates absorption and enhances the analgesic effect of acetaminophen. *The Journal of Clinical Pharmacology*.

[30] Wojcicki J., Rainska-Giezek T., Gawronska-Szklarz B., Dutkiewicz-Serdynska G. (1994). Effects of caffeine on the pharmacokinetics of paracetamol. *Acta Medica et Biologica*.

[31] Boekema P., Lo B., Samsom M., Akkermans L. M., Smout A. J. (2000). The effect of coffee on gastric emptying and oro-caecal transit time. *European Journal of Clinical Investigation*.

[32] Bailey D. G., Dresser G. K., Urquhart B. L., Freeman D. J., Arnold J. M. (2016). Coffee—antihypertensive drug interaction: a hemodynamic and pharmacokinetic study with felodipine. *American Journal of Hypertension*.

[33] Al-Othman A., Al-Musharaf S., Al-Daghri N. M. (2012). Tea and coffee consumption in relation to vitamin D and calcium levels in Saudi adolescents. *Nutrition Journal*.

[34] Gallagher J. C., Yalamanchili V., Smith L. M. (2012). The effect of vitamin D on calcium absorption in older women. *The Journal of Clinical Endocrinology & Metabolism*.

[35] Thom E. (2016). The effect of chlorogenic acid enriched coffee on glucose absorption in healthy volunteers and its effect on body mass when used long-term in overweight and obese people. *The Journal of International Medical Research*.

[36] Ohnaka K., Ikeda M., Maki T. (2012). Effects of 16-week consumption of caffeinated and decaffeinated instant coffee on glucose metabolism in a randomized controlled trial. *Journal of Nutrition and Metabolism*.

[37] Welsch C. A., Lachance P. A., Wasserman B. P. (1989). Dietary phenolic compounds: inhibition of Na+-dependent D-glucose uptake in rat intestinal brush border membrane vesicles. *The Journal of Nutrition*.

[38] Tran A., Zhang C. Y., Cao C. (2015). The role of coffee in the therapy of Parkinson’s disease. *Journal of Alzheimer’s Disease & Parkinsonism*.

[39] Zafar S., Ashraf M. M., Ali A. (2018). Effect of caffeine on anti-clotting activity of warfarin in healthy male albino rabbits. *Pakistan Journal of Pharmaceutical Sciences*.

[40] Rasmussen B. B., Nielsen T. L., Brosen K. (1998). Fluvoxamine is a potent inhibitor of the metabolism of *Caffeinein vitro*. *Pharmacology & Toxicology*.

[41] Jerzy W., Tamara R., Barbara G., Grazyna D. (1994). Effects of caffeine on the pharmacokinetics of paracetamol. *Acta Medica et Biologica*.

[42] Salema B., Ruivo J., de la Torre X., Sekera M., Horta L. (2015). Oxandrolone excretion: effect of caffeine dosing. http://www.adop.pt/media/4114/Oxandrolone_excretion_effect_of_caffeine_dosing_.pdf.

[43] Hartter S., Nordmark A., Rose D. M., Bertilsson L., Tybring G., Laine K. (2003). Effects of caffeine intake on the pharmacokinetics of melatonin, a probe drug for CYP1A2 activity. *British Journal of Clinical Pharmacology*.

[44] Cysneiros R. M., Farkas D., Harmatz J. S., Von Moltke L. L., Greenblatt D. J. (2007). Pharmacokinetic and pharmacodynamic interactions between zolpidem and caffeine. *Clinical Pharmacology & Therapeutics*.

[45] Seeduang C., Manasatienkij C., Rangabphai C., Waiyawuth W. (2013). A study of the in vitro interaction between caffeine and dextromethorphan at high concentrations using human liver microsomes. *Ramathibodi Medical Journal*.

[46] Massey L. K., Whiting S. J. (1993). Caffeine, urinary calcium, calcium metabolism and bone. *The Journal of Nutrition*.

[47] Osswald H., Muhlbauer B., Schenk F. (1991). Adenosine mediates tubuloglomerular feedback response: an element of metabolic control of kidney function. *Kidney International Supplement*.

[48] Zenata O., Marcalíkova A., Vrzal R. (2019). The effect of caffeine on calcitriol-inducible vitamin D receptor-controlled gene expression in intestinal and osteoblastic cells. *Calcified Tissue International*.

[49] Passmore A. P., Kondowe G. B., Johnston G. D. (1987). Renal and cardiovascular effects of caffeine: a dose–response study. *Clinical Science*.

[50] Marx B., Scuvee E., Scuvee-Moreau J., Seutin V., Jouret F. (2016). Mechanisms of caffeine-induced diuresis. *Medical Science*.

[51] Horn J. R., Hansten P. D. (2013). Caffeine and clozapine. *Pharmacy Times*.

[52] Newton R., Broughton L. J., Lind M. J., Morrison P. J., Rogers H. J., Bradbrook I. D. (1981). Plasma and salivary pharmacokinetics of caffeine in man. *European Journal of Clinical Pharmacology*.

[53] Tavares C., Sakata R. K. (2012). Cafeína para o tratamento de dor. *Revista Brasileira de Anestesiologia*.

[54] Yeh J. K., Aloia J. F. (1986). Differential effect of caffeine administration on calcium and vitamin D metabolism in young and adult rats. *Journal of Bone and Mineral Research*.

